# Faculty development in point of care ultrasound for internists

**DOI:** 10.3402/meo.v21.33287

**Published:** 2016-12-13

**Authors:** Anna Maw, Cathy Jalali, Deanna Jannat-Khah, Kirana Gudi, Lia Logio, Arthur Evans, Stacy Anderson, Joshua Smith

**Affiliations:** 1 Department of Hospital Medicine, New York Presbyterian Hospital, New York, NY, USA; 2 Department of Medicine, Weill Cornell Medical College, New York, NY, USA; 3 Department of Medicine, New York Presbyterian-Weill Cornell Medical Center, New York, NY, USA

**Keywords:** point of care ultrasound, faculty development, internal medicine, physical exam, bedside diagnosis

## Abstract

Lack of general medicine faculty expertise is a likely contributor to the slow adoption of point of care ultrasound (POCUS) by internal medicine (IM) residency training programs. We developed a 10-week faculty development program, during which 15 faculty members participated in 2 hours and 10 hours of online didactic and hands-on training, respectively. Pre–post comparisons showed that there were statistically significant improvements in faculty participants' ability to interpret images (*p*<0.001), perceived understanding of the capabilities and limitations of POCUS (*p*=0.003), comfort using POCUS to make clinical decisions (*p*=0.003), and perceptions regarding the extent to which POCUS can improve patient care (*p*=0.026). The next challenge for IM programs is to improve access to ultrasound machines and provide follow-up workshops to facilitate further development of skills and integration of POCUS into daily practice by general medicine faculty.

There is growing evidence that point of care ultrasound (POCUS) has the ability to augment the traditional physical examination and improve patient care by providing real-time, non-invasive, non-radiating, and low-cost imaging to help guide clinical decision-making (1). In response, medical schools around the country are incorporating ultrasound training into their anatomy and physical examination curricula with the expectation that this will better prepare students for their clinical training given the applications of POCUS continue to grow among multiple medical specialties (2, 3). The Critical Care and Emergency Medicine communities were early adopters of this technology and several aspects of POCUS are now considered core competencies in both disciplines. In comparison, internal medicine (IM) has been a slow adopter of POCUS. Among the most important barriers to integration of POCUS into IM residency training is lack of faculty expertise (4).

## Methods

The purpose of this prospective observational study was to develop and evaluate a pilot ultrasound-training program for internist faculty to develop faculty skills in acquiring and interpreting POCUS images.

Over a 10-week period, 15 full-time academic general medicine faculty members at a single urban academic medical center participated in a 12-h (2-h online didactic by SonoSim^®^ and 10 h of small-group hands-on scanning sessions) faculty development course led by an academic hospitalist who had received certification in POCUS exam techniques by the American College of Chest Physicians (A.M.). Hands-on sessions consisted of one to four faculty members performing supervised scanning of medical inpatients, who volunteered to be examined for the purposes of the course and included modules of the diagnostic characteristics and limitations of the POCUS exams most useful for an internist, including vascular, pulmonary, cardiovascular, biliary, and renal. This project was deemed exempt by the WCMC Institutional Review Board.

All faculty participants completed pre- and post-intervention exams and surveys to assess knowledge of and attitudes toward POCUS. The questions centered on test characteristics, image acquisition, interpretation, and clinical correlation of bedside echocardiography including Inferior vena cava (IVC) assessment, lung examination, abdominal (aortic, biliary and renal imaging), and lower extremity Deep Vein Thromobosis (DVT) evaluation.

### Statistical analysis

Given the respondents answers did not follow a normal distribution, a non-parametric test, Paired-Sample Wilcoxon Signed Rank Test, was used to analysis the pre and post test knowledge scores and survey questions addressing faculty attitudes. All statistical tests were 2-tailed and employed an alpha significance level of .05. STATA software (StataCorp. 2013. *Stata Statistical Software: Release 13*. College Station, TX: StataCorp LP) was used to perform all statistical analysis.

## Results

Survey data collected during our pilot study revealed that most participants had no prior training (53%) or only informal training by a colleague (20%) (Table 1). Answers to a 23 question pre- and post-intervention examination showed that faculty had statistically significant improvements in their ability to interpret ultrasound data (*p*=0.0007) (Table 2). Prior to the training session, most faculty members reported minimal to no confidence in their ability to understand and operate the ultrasound equipment (90%). After training, they all reported moderate comfort (*p*=0.0031). Similarly, most trainees had a very limited confidence with respect to understanding the capabilities and limitations of ultrasound, and this attitude was also significantly improved by the training provided (*p*=0.0032) (Table 3).

**Table 1:** Prior training

	Frequency	Percent
Multiple workshops, conferences, or didactics	0	0
At least one workshop conference or didactics	4	26.67
Informal training by a colleague	3	20.00
No prior training	8	53.33

**Table 2:** Pre- and post-test examination scores for attending faculty (*N*=15)

	Pre-test	Post-test	*p*
Median (%)	43.5	87.0	<0.0007
IQR (%)	35–52	74–91	

**Table 3:** Survey data: attitudes and intentions (*N*=11)^a^

Question: To what extent do you think incorporating POCUS into your practice will improve the care you provide to patients?							

	Minimally	Somewhat	Greatly	Not sure		Declined/missing	Wilcoxon signed-rank test

Pre		7 (46.67)	8 (53.33)				0.026
Post		2 (13.33)	13 (86.67)				

Question: To what degree do you agree with the following statement: point of care ultrasound is a diagnostic modality that can be used by clinicians to provide more accurate and quicker diagnosis for several disease processes?							

	Strongly agree	Agree	Neutral	Disagree	Strongly disagree	Declined	Wilcoxon signed-rank test

Pre	5 (33.33)	6 (40.00)					0.10
Post	9 (73.33)	2 (26.67)					

Question: To what degree do you agree with the following statement: Developing skills in point of care ultrasound will help me provide better patient care.							

	Strongly agree	Agree	Neutral	Disagree	Strongly disagree	Declined	Wilcoxon signed-rank test

Pre	6(54.45)	4 (36.36)	1 (9.09)				0.0464
Post	10 (90.90)	1 (9.09)					

Question: How comfortable do you feel with your understanding of the capabilities and limitations of POCUS?							

	Not at all	Minimally	Moderately	Quite	Extremely	Declined/missing	Wilcoxon signed-rank test

Pre	2 (18.18)	8 (72.72)	1 (9.09)				0.0032
Post		1 (9.09)	6 (54.54)	3 (27.27)	1 (9.09)		

Question: How comfortable do you feel with your understanding of the ultrasound machine and your ability to operate it within different clinical scenarios?							

	Not at all	Minimally	Moderately	Quite	Extremely	Declined/missing	Wilcoxon signed-rank test

Pre	6 (54.54)	4 (36.36)	1 (9.09)				0.0031
Post			11 (100)				

Question: How likely are you to integrate POCUS into your routine clinical care?							

	Not likely to use at all	Infrequently	Occasionally	Frequently	Extensively	Declined/missing	Wilcoxon signed-rank test

Pre			5 (45.45)	6 (54.54)			0.59
Post			5 (45.45)	4 (36.36)	2 (18.18)		

Question: Would you recommend this course to your colleagues?							

	Definitely	Probably	Not sure	No			

Post	13 (86.87)	2 (13.33)					

a The first 3 of the 15 faculty members participating in the pilot were not asked the above-mentioned questions.

We asked trainees about perceived barriers to integrating bedside ultrasound into their practice. Pre-test survey data revealed that most trainees identified access to training resources (67%) and lack of time to acquire training (73%) as the greatest obstacles to achieving this goal. Following the training course, a shift in perceived barriers to routine use of bedside ultrasound was revealed: 60% of trainees felt that access to equipment was a limiting factor and lack of time to acquire training remained a persistent concern (67%). Surprisingly, a minority of trainees both before and after the training course (33% and 13%, respectively) had concerns about potential downstream medical–legal liabilities, which could potentially be associated with bedside ultrasound use (Table 3).

Although all participating faculty agreed or strongly agreed that POCUS would improve patient care, the large majority of providers even after the course indicated they plan only occasional or frequent use (verses fully integrated use) of bedside ultrasound for patient examinations. This is perhaps explained by concerns regarding lack of access to equipment and time to acquire more training and practice.

## Discussion

There is substantial interest by IM educators in integrating POCUS curricula into IM training as demonstrated in a national survey conducted in 2012 of IM program directors and assistant program directors which indicated 25% of respondents had a formal curriculum for residents and 25% planned on starting one within 12 months (5). Despite this interest in curriculum development, there are currently no standards for training in IM POCUS and there are still many questions regarding how much training is sufficient to competently interpret images and make clinically sound decisions based on bedside ultrasound findings (6). Most IM residency POCUS programs consist of isolated workshops, didactics, and simulation (5–7). There is none currently described that consists of full integration of POCUS into the routine bedside evaluation of patients on a daily basis with expert supervision which is the traditional and principal method by which trainees develop clinical skills during residency. This emphasizes the importance of providing teaching faculty with training that ensures competence, as without it, our attempts to adequately train residents in POCUS will likely fall short.

Our own pilot study showed that a brief intensive course that includes didactics and hands-on training yielded significant improvements in the ability of our faculty to obtain and interpret ultrasound data as well as change faculty attitudes regarding the utility of this new tool. Our survey data also reflect an appropriately perceived lack of mastery post-intervention by participants. Currently, there are few opportunities for internists to pursue POCUS training. While we feel that our pilot study brings to light several barriers with respect to integration of POCUS in IM Programs, our study involved a small group of self-selected faculty (*N*=15). Although faculty clearly benefitted from this brief intensive training course, it remains unclear how long this higher level of skill will be maintained in the absence of continued training.

Our long-term goal is to develop a rigorous training program for faculty that ensures competency by offering longitudinal mentorship over an extended period of time with regular assessments of competency post-training. This program would likely consist of many hours of didactics and supervised hands-on scanning followed by submission of a video portfolio consisting of a minimum number of recorded exams deemed adequate in technique by expert ultrasound faculty as well as a written and practical examination following a several month long curriculum.

Some clinicians may be uneasy about basing medical decisions on non-expert bedside ultrasound examinations. It may be true that compared to a certified experienced technician, a POCUS novice will produce tests with lower sensitivity and specificity (8–10). However, some research has shown that this is not always the case (11). We advocate that bedside ultrasound should not be considered an alternative to formal technician-acquired radiologist-interpreted ultrasound studies, and its test characteristics should not be compared with these exams (12). The comparison should instead be made to other traditional bedside physical examination tools such as the stethoscope, penlight, reflex hammers, etc. By that standard, there is little question that POCUS augments the diagnostic power of the traditional physical examination and should become an integrated part of the physician's bedside armamentarium. For example, in a patient presenting to the clinic or emergency department with dyspnea, in addition to traditional physical examination maneuvers including auscultation of the lungs and heart as well as evaluation of the neck veins to detect jugular venous distention, the physician might also perform a lung ultrasound exam to evaluate for signs of pneumonia, pulmonary edema or pleural effusion, all entities for which ultrasound has been shown to be as or more accurate than portable chest radiograph (13–16). In addition, the clinician might also evaluate for left ventricular systolic dysfunction and elevated central venous pressure using ultrasound. In a well-trained provider, the addition of the POCUS evaluation would add considerable diagnostic value and add only a few minutes to the patient encounter (17).

For the vast majority of providers, bedside ultrasound improves their ability to provide patient care. The challenge for IM departments is the development of a training program that ensures competency in the use of this powerful diagnostic tool. Our preliminary data show that a brief intensive training course yields significant improvements in operator acquisition and interpretation of ultrasound data. Our findings suggest that exposure is key in developing skills as well as an increased awareness of POCUS as a tool in the armamentarium of providers. This emphasizes a need for faculty development in understanding and incorporating new technologies into clinical practice. Internal Medicine Departments will need to develop a mechanism to provide structured training in this innovative clinically relevant technology to faculty in order to ensure their competency and thus ensure adequate training of residents. We plan to expand our own curriculum to include longitudinal mentored training that creates a definitive pathway for POCUS mastery by IM faculty.
